# Effects of Sachet Water Consumption on Exposure to Microbe-Contaminated Drinking Water: Household Survey Evidence from Ghana

**DOI:** 10.3390/ijerph13030303

**Published:** 2016-03-09

**Authors:** Jim Wright, Mawuli Dzodzomenyo, Nicola A. Wardrop, Richard Johnston, Allan Hill, Genevieve Aryeetey, Richard Adanu

**Affiliations:** 1 Geography and Environment, University of Southampton, Southampton SO17 1BJ, UK; Nicola.Wardrop@soton.ac.uk; 2 Ghana School of Public Health, University of Ghana, Accra PO Box LG13, Ghana; mdzodzo@hotmail.com (M.D.); cecearyeetey@yahoo.co.uk (G.A.); rmadanu@yahoo.com (R.A.); 3 Joint Monitoring Programme for Water Supply and Sanitation (JMP), Department of Public Health, Environmental and Social Determinants of Health (PHE), World Health Organization, Geneva 1211 Switzerland; johnstonr@who.int; 4 Social Sciences, University of Southampton, Southampton SO17 1BJ, UK; ah4e10@soton.ac.uk

**Keywords:** drinking water, beverages, Escherichia coli, West Africa, Survey methodology

## Abstract

There remain few nationally representative studies of drinking water quality at the point of consumption in developing countries. This study aimed to examine factors associated with *E. coli* contamination in Ghana. It drew on a nationally representative household survey, the 2012−2013 Living Standards Survey 6, which incorporated a novel water quality module. *E. coli* contamination in 3096 point-of-consumption samples was examined using multinomial regression. Surface water use was the strongest risk factor for high *E. coli* contamination (relative risk ratio (RRR) = 32.3, *p* < 0.001), whilst packaged (sachet or bottled) water use had the greatest protective effect (RRR = 0.06, *p* < 0.001), compared to water piped to premises. *E. coli* contamination followed plausible patterns with digit preference (tendency to report values ending in zero) in bacteria counts. The analysis suggests packaged drinking water use provides some protection against point-of-consumption *E. coli* contamination and may therefore benefit public health. It also suggests viable water quality data can be collected alongside household surveys, but field protocols require further revision.

## 1. Introduction

Given continued population growth, utilities in many cities in sub-Saharan Africa struggle to provide sufficient domestic water to meet residents’ needs [1]. The gap between supply capacity and demand manifests itself through water rationing, with supply interruptions common in many urban neighbourhoods [2]. Faced with water rationing, residents not only have to find alternative sources of water when piped supplies are unavailable and store water to prepare for interruptions, but they may also be exposed to water contamination events associated with pressure drops within the supply system [3]. Furthermore, stored water may often become contaminated [4], and recent systematic review evidence suggests the extent of stored water contamination is greater among those using piped supplies [5].

A recent nationally representative household survey in Ghana, the Living Standards Survey Round 6 (GLSS6), was conducted in 2012–2013 and included an additional module on drinking water quality. Although some household surveys have previously collected water quality parameters [6], this was the first nationally representative survey published which tested for a microbiological water quality parameter. The GLSS6 [7] suggested that rates of detectable *E. coli*, routinely used to indicate the presence of faecal contamination, were lower in sachet water than in piped water, consistent with low microbial contamination in packaged water relative to many other sources in a recent systematic review [8]. However, more in-depth analyses of the water quality data from this survey, examining patterns of microbial contamination while controlling for important confounding factors (e.g., water storage practices), have not yet been conducted. 

In many sub-Saharan African cities, packaged waters have emerged as an alternative source of drinking water, both for those with piped connections and those without. In urban Ghana, consumption of “sachet” water (water sold in 500 mL sealed plastic bags) has grown rapidly [9]. Bottled water is also available, but less commonly used as a main source of drinking water. Larger corporate sachet producers and smaller producers registered with the regulatory bodies, the Ghana Standards Authority and Food Standards Authority, typically use pre-filtration, ultra-violet and reverse osmosis treatment as part of their production processes. However, unregistered producers also exist whose production processes have not been subject to regulatory scrutiny [9], and who often produce cheaper sachets to undercut more established sachet brands. It has been suggested that those living in poorer neighbourhoods may be particularly exposed to these lower quality brands [10], with associated concerns around sachet water safety.

This study therefore draws on GLSS6 data to examine factors associated with microbial contamination of point-of-consumption drinking water. In doing so, it seeks to examine the plausibility of microbial contamination patterns from the new household survey water quality module. As a secondary objective, the study examines risk factors for packaged (sachet and bottled) water contamination, both at the point of consumption and point of sale, while controlling for confounders. It aims to assess whether poorer households are differentially exposed to microbially contaminated packaged water. It also aims to quantify the protective effect, if any, of packaged water against point-of-consumption microbial contamination. 

## 2. Experimental Section

### 2.1. Data

The GLSS6 is a multi-stage cluster survey designed to generate representative estimates for Ghana’s 10 regions and ecological zones, which was conducted from October 2012 to October 2013. Within the survey, 15 households were selected in each of the 1200 enumeration areas (EAs), giving a total of 18,000 households. Three households from each cluster of 15 (3600 households in total) were randomly selected and asked to provide “a glass of water which you would give a child to drink” [7,11] and a water sample was taken from this glass. A random number generator was used to select three households per cluster, so household randomization to water testing was constrained by survey cluster. For one in three of these selected households (1200 households in total), a second sample was taken from the source of water. In the case of packaged waters, the water would thus be poured into a glass or other vessel when sampling water at the point of consumption, whilst water sampled directly from packaging acted as source water samples (*i.e*., without transfer to a drinking vessel). These samples were then tested for arsenic, total coliforms and *E. coli* only. Microbial testing took place in the field, with 100 mL of sample water being filtered through a 0.45-micron filter. This was then placed onto a Compact Dry EC media plate (Nissui, Japan) and incubated at ambient temperature for 24 h. An additional 1 mL of water from each sample was placed on a second media plate without filtering, and GLSS6 field staff counted total coliform and *Escherichia coli* colonies on both 1 mL and 100 mL plates. Additional quality assurance measures were undertaken for a subset of households. These measures included duplicate laboratory testing for 10% of water samples, as well as analysis of sterile “blanks” for *E. coli* in the field alongside 5% of samples taken. Laboratory testing was undertaken via the regional laboratory network of the main utility, the Ghana Water Company. Laboratory testing methods differed from the survey method and varied between laboratories, with most using a Most Probable Number method, two using membrane filtration methods, and one using a presence-absence method. However, some aspects of duplicate laboratory testing proved problematic. This was because field sites were often distant from laboratories and therefore sample transit times exceeded 6 h, but also when samples did reach laboratories, limited laboratory capacity sometimes meant samples could not be processed in a timely fashion. Colony-forming unit (cfu) counts greater than 100 were considered too numerous to count.

### 2.2. Preliminary Evaluation of E. coli Counts

Histograms of the observed cfu counts per sample were plotted to assess the distribution of 1 mL and 100 mL samples at both source and at the point of consumption. Since there was evidence of digit preference (the tendency to round values to pleasing numbers) in all four of these histograms, end-digit frequencies were also calculated and plotted. Digit preference in data such as these is often considered a marker of authenticity. However, digit preference results in unusual data distributions, with implications for the most appropriate regression approach in further analysis.

### 2.3. Assessing Risk Factors for Water Contamination at the Point of Consumption

To address the aims of this analysis, the relationships between source types, household socio-economic status (SES) and *E. coli* contamination of water were assessed because of *E. coli*’s advantages over total coliforms as a biological indicator for public health protection [12,13]. As bottled water represents a minor source of drinking water in Ghana (only 4 of the 3096 samples here were from bottled water), this water source was combined with sachet water to give a “packaged water” category. Systematic review evidence suggests microbiological contamination varies even within “improved” source types. Given also that there were few samples for source types not in widespread use (e.g., tankers), water sources were therefore grouped into the following categories: (a) piped to dwelling (including piped into home and piped to yard/plot); (b) standpipe, water tanker or piped to neighbour; (c) borehole; (d) protected well; (e) unprotected well or spring; (f) rainwater collection; (g) surface water collection; (h) packaged water (sachet or bottled water). Prior to grouping source types, we checked for similarity in contamination patterns. During univariate analysis, more detailed categories for borehole sources were also included (community-provided boreholes; boreholes provided by non-governmental organizations (NGOs), and boreholes managed in other ways), to allow assessment of different water providers in the contamination level of water from boreholes, given growing policy interest in the effectiveness of state *versus* private sector water provision [14] and in the effectiveness of community- or household-led self-supply [15]. As noted, it has been suggested that poorer households may be more likely to consume poorer quality, cheaper counterfeit packaged water brands distributed by unregistered producers [10]. To examine exposure among poor households, expenditure (regionally deflated total expenditure (GH₵) per day, per adult equivalent) was used in preference to income as a measure of SES because consumption data are less subject to short-term fluctuations than income, since households prefer to smooth consumption by holding back a portion of income as savings [16].

Access to improved sanitation (including water closet, pit latrine, Kumasi ventilated improved pit latrine (KVIP), and public toilet; shared facilities of these types were also considered as improved) and the presence of soap or detergent within the household were examined in relation to water contamination, given the plausible link between poor sanitation, handwashing behaviours and contamination of drinking cups or stored water [4]. Information on if, and how, water was stored prior to sampling was assessed (categorised as not stored (*i.e*., obtained directly from source, including directly from sachet), stored in a covered vessel or stored in an uncovered vessel), as numerous studies have shown water storage in the home to be associated with contamination [4,5]. The effect of urban *versus* rural location was included as there has been debate as to whether drinking water contamination risks are inherently greater in urban areas and whether, accordingly, different urban monitoring arrangements should be adopted [17]. Finally, groundwater sources were differentiated from other sources and categorised as being either an unsafe distance from a latrine (less than 30 m) or beyond this threshold, following standard sanitary risk inspection guidance for sources such as protected wells and springs [18]. Home water treatment was not assessed, as water treatment information was only requested from households reporting noticeable organoleptic issues with their water (e.g., odour, colour), thus resulting in a large number of missing data for this variable.

As noted above, initial examination of microbiological data suggested pronounced digit preference, making it problematic to identify an appropriate statistical distribution on which to base any subsequent analysis. Therefore, *E. coli* cfu data were categorised and multinomial logistic regression was used to examine risk factors for water contamination at the point of consumption, adjusting for the survey design via the *svy* commands in Stata 13. Household weights (the reciprocal of the probability of household selection) were used, given the focus on household level variation in contamination of drinking water. The cfu data based on 100 mL samples only were used, as these are likely to be less uncertain than results from a smaller (1 mL) water sample. Initially, univariable analysis was used to assess the relationships between hypothesised risk factors and water contamination. A *p*-value of 0.05 or smaller was considered to be statistically significant. Subsequently, covariate selection for multivariable regression was carried out using the results of univariable analysis, assessment of collinearity between variables and prior understanding of potential risk factors in water contamination. Water source and expenditure were included in the multivariable analysis as covariates of primary interest. The presence of effect modification was assessed by inclusion of interaction terms between expenditure and water source.

The sensitivity of the multivariable regression model results to the removal of potentially erroneous water contamination observations was tested. Because of digit preference, it proved problematic to assess the underlying bacterial density distribution in these data and thereby identify statistically significant differences between pairs of 1 mL *versus* 100 mL samples. Therefore, using a “rule of thumb”, observations were removed where (a) any level of contamination was detected in the 1 mL sample, with less than 10 cfu detected in the 100 mL sample; (b) more than 2 cfu was detected in the 1 mL sample and less than 30 cfu in the 100 mL sample; or (c) contamination levels were greater in the 1 mL sample than the 100 mL sample. This reduced the overall sample size from 2822 to 2552. The final multivariable model, including interaction terms, was then fitted using the smaller dataset.

### 2.4. Risk Factors for Purchasing of Contaminated Sachet Water

Considering water samples taken directly from source, only 26 of 132 sachet samples (bottled water was not considered in this analysis) had detectable contamination in 100 mL. Therefore, we used logistic regression to examine three potential risk factors for the purchase of sachets contaminated with *E. coli* in 100 mL samples, adjusting for the survey design. Regionally deflated total expenditure per adult equivalent household member was assessed as an indicator of household purchasing power, since it has been suggested that poorer households may be more likely to buy lower quality brands [10]. We also examined the impact of region and rurality on contamination rates, to assess whether there was evidence for geographic variation in sachet safety.

## 3. Results

### 3.1. Digit Preference in Bacterial Counts

**Figure 1:**
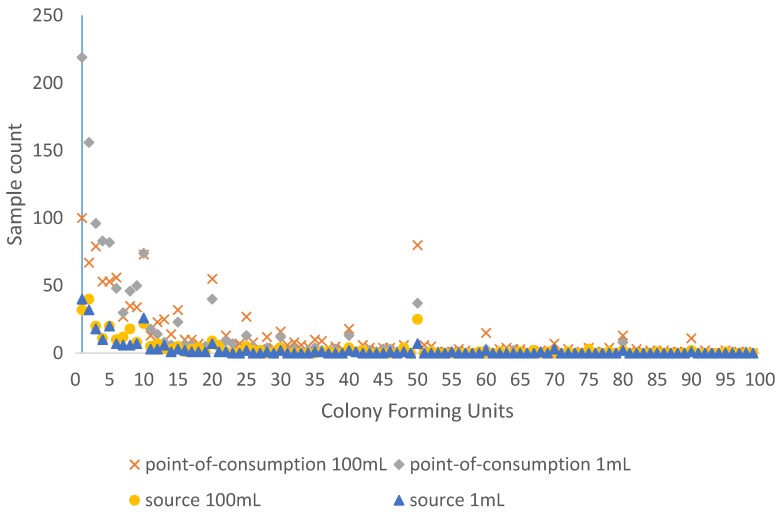
Distribution of *E. coli* colony-forming unit (cfu) counts from membrane filtration in the range 1 to 99cfu for 1 mL and 100 mL point-of-consumption and source water samples.

Figure 1 shows the frequency distribution of the point-of-consumption and source 1 mL and 100 mL membrane filtration cfu counts between 1 and 99. “Heaping” of samples was apparent, with many samples having cfu counts ending in a zero (e.g., 10, 20, *etc.*) or a five, particularly as the counts grew larger. 

**Figure 2:**
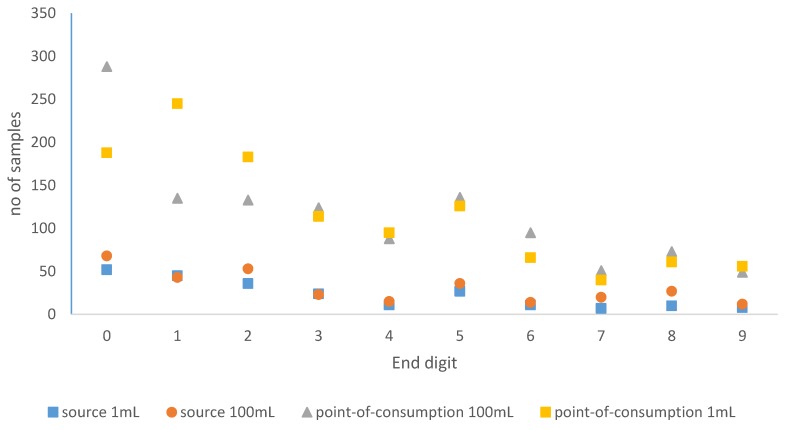
Distribution of end digits in *E. coli* colony forming unit (cfu) counts between 1 and 99 from membrane filtration of 1 mL and 100 mL source and point-of-consumption samples.

This pattern is confirmed by Figure 2, which shows the frequency of end digits in observed cfu counts, including only those samples with cfu counts in the range 1 to 99 (to avoid any impact of non-detects and too numerous to count (TNTC) samples). Even excluding samples with no detectable *E. coli* and TNTC samples, zero is by far the most popular end digit, with an apparent secondary peak in the number of samples with counts ending in five.

### 3.2. Risk Factors for Contaminated Water at Point of Consumption

Allowing for household non-response and other protocol deviations, water from 3096 households was tested at the point of consumption and 1066 households at source. Table 1 shows the distribution of point-of-consumption *E. coli* contamination (weighted percentages) in relation to a range of household characteristics, weighted by household. Given the “heaping” of sample results at 10 cfu/100 mL and 100 cfu/100 mL, we assessed risk factors for medium (1 to 72 cfu/100 mL) and high (>72 cfu/100 mL) *E. coli* contamination of drinking water at the point of consumption in 100 mL samples. The boundary between these classes thus avoided the “heaped” values in the cfu count distribution in Figure 1 above, and ensured roughly equal numbers of observations within each of the contamination categories. Having equal numbers reduced subsequent regression model instability arising from small cell counts in a cross-tabulation of contamination level against sample characteristics.

Relative risk ratios from univariable multinomial logistic regression analysis for each potential risk factor are shown in Table 2. Based on univariable analysis, packaged water use decreased the risk of medium or high contamination with *E. coli* at the point of consumption compared to water piped to the premises (relative risk ratio (RRR) = 0.30 for medium contamination and RRR = 0.06 for high contamination; *p* < 0.001 for both). All other water source categories were associated with increased risk (RRR > 1) of medium and high contamination at the point of consumption. Increasing expenditure (regionally deflated GH₵ per day, per adult equivalent) was associated with reduced risk of medium or high levels of contamination at the point of consumption (RRR = 0.97 for medium contamination and RRR = 0.89 for high contamination; *p* < 0.001 for both). RRRs for expenditure relate to a one GH₵ increase in expenditure, equivalent to approximately GB£ 0.17 or US$ 0.25. The availability of improved sanitation and soap, and residence in an urban area (compared to rural) were all associated with lower risk of contamination at the point of consumption (see Table 2). In comparison to obtaining water directly from the source, water obtained from a covered storage vessel or an uncovered storage vessel had higher risk of contamination (see Table 2).

The number of covariates included in the multivariate multinomial regression analysis had to be reduced due to collinearity between variables. Water source and expenditure were included as variables of primary interest. Improved sanitation and whether the water sample was obtained from source, or from a covered or uncovered storage vessel, were included as potential confounding factors, due to their implications for water contamination. The presence of soap was not included due to collinearity with improved sanitation; urban *versus* rural was not included due to collinearity with water source and other variables such as improved sanitation; water storage container was not included due to collinearity with water source; and distance between latrine and water source was not included as the main effect seen here was the protective effect of non-ground water rather than a difference in contamination depending on the distance. An interaction effect was included between expenditure and water source, to examine any SES-related differences in the relationship between sachet water use and water contamination at the point of consumption.

After controlling for confounding between measured household characteristics, use of packaged water (relative to water piped to the premises) had a protective effect against medium and high levels of *E. coli* contamination of point-of-consumption samples (RRR = 0.18 for medium contamination and RRR = 0.04 for high contamination; *p* < 0.001 for both; see Table 3). Surface water use was associated with the largest risk of high levels of *E. coli* contamination of samples (RRR = 42.01, *p* < 0.001). In addition, use of protected wells (RRR = 9.32, *p* = 0.004), unprotected wells or springs (RRR = 10.27, *p* < 0.001), boreholes (RRR = 6.36 (community operated); RRR = 6.86 (NGO operated); 8.68 (other providers); *p* < 0.01 for each) and standpipes, tanker water or water piped to a neighbour’s home (RRR = 8.37, *p* < 0.001) were significant risk factors for high levels of *E. coli* contamination at the point of consumption; and use of water from standpipes, tankers or water piped to a neighbour’s home (RRR = 2.37, *p* = 0.02) or NGO-operated boreholes (RRR = 2.85, *p* = 0.05) were also significant risk factors for medium levels of contamination. Increased expenditure was correlated with a decreased risk of medium or high level contamination, although after inclusion of an interaction term, the main effects were not statistically significant (RRR = 0.98, *p* = 0.08 for medium contamination and RRR = 0.98, *p* = 0.22 for high contamination). There was a significant interaction effect between expenditure and the surface water use for high contamination, suggesting that the protective effect of increasing expenditure was stronger for households using surface water sources (RRR for the interaction effect = 0.89, *p* = 0.009). There were no significant interaction effects detected for the other water source categories. Improved sanitation was not statistically significant in the multivariable model (RRR = 0.82, *p* = 0.26 for medium contamination and RRR = 0.90, *p* = 0.56 for high contamination). Water taken from an uncovered storage vessel (*versus* directly from source) was a significant risk factor for high levels of *E. coli* contamination at the point of consumption (RRR = 2.82, *p* = 0.03); while water taken from a covered storage vessel was protective of high levels of contamination (RRR = 0.60, *p* = 0.02). 

The removal of potentially erroneous observations reduced the dataset to a sample size of 2647 (from 2972). The model parameters using the reduced dataset are shown in supplementary information Table S1. In brief, the results altered as follows: improved sanitation and water obtained from community-operated boreholes were significantly associated with the risk of medium contamination (RRR for improved sanitation = 0.69, *p* = 0.05; RRR for community-operated boreholes = 3.33, *p* = 0.002), and obtaining water from an uncovered storage vessel was not significantly correlated with high levels of contamination using the reduced dataset (RRR for water from an uncovered vessel = 1.93, *p* = 0.12). In addition, there was a significant interaction between expenditure and NGO-operated boreholes (RRR for the interaction effect = 0.89, *p* = 0.04), indicating that the protected effect of increased expenditure against medium-level contamination was stronger for water obtained from NGO-operated boreholes. The relationships between contamination levels and other covariates remained broadly unchanged, although confidence intervals were wider due to the smaller sample size. We also explored the impact of introducing an additional low contamination banding into our multivariate analysis of *E. coli* counts (Tables S2–S4). Results were similar to the analysis with fewer contamination bandings.

### 3.3. Risk Factors for Purchasing Contaminated Sachet Water

Based on the results of multivariable logistic regression analysis, the presence of detectable *E. coli* contamination of sachet water (where samples were taken directly from the source, the sachet itself) was not significantly associated with rural *versus* urban areas, geographical region or expenditure (*p* > 0.05; Table 4). Overall, 19.7% of “source” sachet samples contained detectable *E. coli*.

## 4. Discussion

This analysis suggests that *E. coli* contamination patterns in the GLSS6 are plausible and broadly consistent with evidence from elsewhere. For example, systematic review evidence suggests household stored water is often more contaminated than water taken directly from the source [4,5], and in the GLSS6, uncovered stored water was associated with higher contamination (Table 2 and Table 3). The relative extent of *E. coli* contamination across the different source types is also broadly consistent with recent systematic review evidence [8], in that there was some contamination even among “improved” sources such as piped supplies and boreholes. 

The findings also broadly support the WHO/UNICEF Joint Monitoring Programme (JMP) “ladder,” which differentiates surface water from other unimproved sources such as unprotected wells, and piped water to the premises from other forms of improved supply. This “ladder” forms the basis for proposed post-2015 monitoring of differing levels of service access, over and above “improved” *versus* “unimproved” water sources [19]. In the GLSS6 data, surface water was associated with the greatest risk of high levels of *E. coli* contamination at the point of consumption, whilst contamination risks were higher for standpipes than for water piped to the premises (Table 3). There was no evidence of borehole contamination differences between community-managed, NGO-managed and other arrangements.

The analysis suggests that among measured behaviours, packaged water use (which is predominantly sachet water) afforded the greatest protective effect against high levels of *E. coli* contamination of water at the point of consumption (Table 2 and Table 3). Use of packaged water reduced risk of *E. coli* contamination, even relative to use of piped water onto premises. Sachet samples taken directly from packaging were less contaminated than those taken from drinking cups, supporting recent work suggesting sachet contamination increases between point of manufacture and point of sale [20]. This is consistent with some evidence that packaged water has a protective effect against child diarrhoea [2]. Furthermore, since many households stored water in sachets (Table 1), it is plausible that the sachets protected against recontamination from handling and since, unlike bottles, sachet packaging cannot be reused, this may offer further protection against recontamination. This apparent protective effect of packaged water against contamination with *E. coli* suggests that restricting use of sachets (for example via a ban proposed in Ghana in 2007 as reported in [9]) could potentially have consequences for public health.

However, in absolute terms, over 30% of sachet samples tested positive for *E. coli*, a proportion greater than that reported in any of the sachet water studies included in a recent systematic review [8].

Packaged water is often drunk directly from the packaging, although the water can also be decanted into a glass or bottle (e.g., for consumption by a child): thus, the method of sampling from a glass may not be as representative of the conditions at the point of consumption as the other water source categories which would mostly be consumed from a glass. This is unlikely to impact the overall results, although there is the potential for overestimation of point-of-consumption contamination of packaged water due to contamination of the drinking vessel used to provide the sample.

There is no evidence here that poorer households are differentially exposed to contaminated packaged water (Table 1) as hypothesised previously [10], though there is evidence that households with low expenditure using surface waters are more exposed to contaminated water at the point of consumption (Table 3). This latter finding supports the suggestion that socio-economic inequality in safe water access may be even more pronounced if water quality is taken into account in addition to water source type [6].

The membrane filtration results presented here show evidence of digit preference (Figure 1 and Figure 2), which may reflect the field-based rather than laboratory-based testing undertaken. Whilst there is a long history of examination of digit preference to assess the quality of demographic data [21] and clinical data such as blood pressure readings [22], there are few if any published studies examining digit preference in microbiological data from water samples. In the absence of other studies of digit preference in water microbiology data, we cannot ascertain whether the problem is particularly pronounced in this dataset. This apparent digit preference in cfu counts has a number of implications. Many studies (e.g., [23,24,25,26] report water quality data in risk bandings of 1–10 or 1–9 cfu/100 mL, 11−100 or 10–99 cfu/100 mL, and greater than 99 or 100 cfu/100 mL. Despite this banding, systematic reviews have found no evidence for increased risk of diarrhoeal disease as cfu/100 mL values increase above 1 cfu/100 mL [25,27], so these categories are essentially arbitrary. Where there is digit preference because those interpreting membrane filtration results round cfu counts to pleasing numbers, these arbitrarily chosen risk interval boundaries may be inappropriate. This is because the interval boundaries fall at the “heaped” values of 10 and 100 and these rounded values will all be assigned to either the higher or lower class. Choosing alternative risk band boundaries that do not end in zero would avoid this issue, as would using smoothing or related techniques [28] to redistribute “heaped” values prior to reporting. As in demography and clinical medicine, it may be that analysis of end digits could be used more widely as a quality control measure in examining membrane filtration results. 

These findings are subject to several sources of uncertainty, most notably with the field-based testing for *E. coli*. Problems have been noted with the field implementation of the GLSS6 [11]. For example, some of the quality control measures were inconclusive because of inconsistent recording of “blank” sample results and a lack of capacity in laboratories scheduled to undertake duplicate testing. In addition, consistency checks between the 100 mL and 1 mL samples resulted in the exclusion of approximately 10% of the overall sample. This may be due to errors in the testing procedure or recording of results, although assumed inconsistencies between the two samples may also arise due to chance, particularly where the level of contamination is low. The regression results did not change substantially after removing these potentially erroneous results. 

More generally, this analysis focused only on *E. coli* as faecal indicator bacteria, but the relationship between indicator bacteria and pathogen presence is complex [29]. One review [30] found that *E. coli* counts were correlated with intestinal pathogens in only 11 out of 40 studies of recreational or drinking waters. Moreover, there is evidence from tropical environments that *E. coli* can originate from non-faecal sources [31], and that its regrowth in such environments can be affected by parameters such as soil moisture [31]. On the other hand, *E. coli* may be attenuated or inactivated in the environment more rapidly than some pathogens, so the absence of *E. coli* does not guarantee the absence of pathogens. In part for these reasons, epidemiological evidence linking diarrhoeal disease risk to *E. coli* in drinking water is mixed. Despite some studies showing no apparent relationship, a meta-analysis found a pooled association between diarrhoea and *E. coli* presence from 14 studies [27]. Thus, whilst for logistical and budgetary reasons a household survey module necessarily has to concentrate on a very limited set of water quality parameters, *E. coli* remains the principle recommended water quality parameter [13]. Because of the need to conduct tests in remote locations lacking laboratory infrastructure, Compact Dry EC media were used to enumerate *E. coli* rather than a standard method. Although this is one of 20 products identified as suitable for *E. coli* enumeration in remote or resource-poor settings [32], there remain few comparative evaluations of such field methods and none of the Compact Dry EC media. 

The small number of “source” samples taken directly from sachets (*i.e.* without transferring to a drinking vessel) limits any ability to detect differences in exposure among rich *versus* poor sachet users. Similarly, the small proportions of households practicing behaviours such as home water treatment also limit our ability to detect their water quality impacts. Confounding may also affect this cross-sectional survey. Sachet use is predominantly an urban phenomenon, particularly in Greater Accra, and may be associated with other unmeasured factors that protect against point-of-consumption water contamination in urban areas. Several potential predictors of contamination, such as piped supply interruptions [3], residual-free chlorine [5,8], the characteristics of the cup or glass used to serve water [33] and sachet water brands [10], were either not recorded in the GLSS6 or recorded too inconsistently to be usable. Although sachet use is less common in rural areas, univariate analysis indicated that the quality of sachet water in rural areas was comparable to, or better than, the quality of sachet water in urban areas (67% of sachet samples in urban areas and 81% of sachet samples in rural areas were uncontaminated by *E. coli*). There are some additional influences on water contamination not considered in this manuscript, notably rainfall patterns [34] and seasonality. There would be some potential to expand the set of risk factors for contamination considered in this analysis, though this would require spatial linkage to gridded rainfall and other datasets and the lack of detailed spatial representation in the GLSS6 somewhat restricts such analysis. 

Given these issues, we propose several revisions to any future water quality module implemented alongside a household survey. Firstly, field-based quality control measures should be consistently implemented and recorded. Field teams should routinely analyse field blanks (water known to be free of *E. coli* contamination), with at least one blank per ten actual tests. Secondly, where non-technical staff are conducting the water quality test, they should be supported by local water quality laboratory workers. These laboratory workers should at a minimum participate in training sessions, and if possible also visit field teams during data collection to ensure that field staff are correctly and consistently following standard operating procedures. Ideally, a subset of duplicate samples should be sent on ice to laboratories for cross-checking analysis, within 24 h of collection. If this is logistically infeasible, a smaller number of samples collected from sites close to the laboratories could be cross-checked. Finally, data quality checks should be implemented alongside data collection (including comparison of 1 mL and 100 mL sample results, and examination of digit preference patterns), to identify any substantial variation between different testing teams and to take appropriate corrective measures while field work is still underway. A further challenge to be addressed is the identification of an appropriate statistical distribution for a given bacterial density dataset, where digit preference exists. This would enable the subsequent identification of statistically significant differences in replicate sample results as an additional quality control measure.

## 5. Conclusions

Our analysis suggests that microbial data collected through a nationally representative household survey module follow plausible patterns and provide evidence to support the current “ladder” of water source types used in international monitoring of safe water access. Some revisions to the survey module protocol are however recommended. Relative to household use of water piped to the premises, use of packaged water had a large protective effect against high levels of *E. coli* contamination at the point of consumption. This suggests that from a public health perspective, policy measures that seek to limit packaged water consumption, such as the outright bans that have sometimes been proposed, could potentially increase population exposure to microbial contamination. However, even among sachets, over 30% of point-of-consumption samples tested positive for *E. coli*, suggesting a need for greater regulatory oversight of this industry and safer sachet handling in the home. Our analysis also identifies the presence of digit preference in microbial data, which—if present in other membrane filtration datasets—has implications for the way such data are analysed and presented.

## Figures and Tables

**Table 1:** Descriptive statistics for *E. coli* contamination of water at the point of consumption, *n* = 2972.

Variable	Category	Contamination Level * (Rows Sum to 100%)			*n*
		Uncontaminated	Medium	High	*n*
Total		28.0%	37.3%	34.7%	2972
Source contamination risk factors					
Water source	Piped to premises	34.5%	49.2%	16.4%	240
	Standpipe, tanker or neighbours tap	13.8%	42.2%	44.0%	500
	Protected well	11.8%	31.1%	57.2%	101
	Unprotected well or spring	12.2%	36.9%	50.9%	142
	Rainwater	7.1%	51.4%	41.6%	57
	Surface water	5.1%	17.1%	77.8%	315
	Packaged water	68.8%	29.2%	2.0%	590
	Community borehole	10.7%	44.1%	45.2%	565
	NGO borehole	13.6%	41.9%	44.6%	207
	Other borehole	7.9%	42.7%	49.4%	218
	Missing	9.7%	64.5%	25.9%	37
Distance between latrine and water source	Within 30 m	8.6%	38.2%	53.2%	488
	More than 30 m	10.0%	36.3%	53.7%	1097
	Not ground water	42.5%	37.6%	19.9%	1387
Urban	No	15.5%	36.0%	48.6%	1667
	Yes	38.4%	38.4%	23.3%	1305
Recontamination risk factors					
Improved sanitation	No	13.6%	37.6%	48.8%	2076
	Yes	31.5%	37.1%	31.5%	890
	Missing	6.3%	73.0%	20.7%	6
Soap observed	No	19.3%	38.7%	42.1%	1765
	Yes	32.5%	36.6%	30.9%	1207
Water obtained from source or vessel	From source	47.1%	33.1%	19.8%	891
	From covered vessel	17.4%	41.5%	41.1%	1667
	From uncovered vessel	6.5%	27.6%	65.9%	295
	Missing	23.4%	41.1%	35.5%	119
Water storage container	Plastic bucket or container	24.7%	37.7%	37.7%	1963
	Pot or earthenware vessel	7.6%	41.6%	50.7%	499
	Metal container	12.7%	30.3%	57.1%	137
	Other (including sachets)	61.7%	34.1%	4.2%	361
	Missing	59.8%	40.2%	0%	12
Expenditure (GH₵)	Mean	13.3	9.0	6.5	NA
	Median	9.0	6.7	4.9	NA
	Range (min–max)	0.70−227.9	0.33−103.3	0.18–60.1	NA

* Contamination levels: uncontaminated (0 cfu/100 mL); medium contamination (1 to 72 cfu/100 mL); high contamination (>72 cfu/100 mL).

**Table 2:** Relative risk ratios for medium (1 to 72 cfu/100 mL) and high (>72 cfu/100 mL) contamination of water at the point of consumption with *E. coli* derived from univariable multinomial regression analysis.

Covariate	*n*	Category	Medium Contamination (1 to 72 CFU)		High Contamination (73+ CFU)	
			RRR * (95% CI)	*p*-value	RRR * (95% CI)	*p*-value
Water source	2935	Piped to premises	Reference		Reference	
		Standpipe, tanker or neighbours tap	2.14 (1.26 to 3.64)	0.005	6.69 (3.57 to 12.55)	<0.001
		Protected well	1.85 (0.76 to 4.50)	0.17	10.24 (4.30 to 24.39)	<0.001
		Unprotected well or spring	2.11 (0.66 to 6.74)	0.21	8.76 (2.67 to 28.76)	<0.001
		Rainwater	5.10 (1.43 to 18.15)	0.01	12.38 (3.13 to 48.94)	<0.001
		Surface water	2.36 (1.02 to 5.49)	0.05	32.28 (13.63 to 76.48)	<0.001
		Packaged water	0.30 (0.18 to 0.49)	<0.001	0.06 (0.03 to 0.13)	<0.001
		Community-managed borehole	2.88 (1.62 to 5.15)	<0.001	8.89 (4.68 to 16.88)	<0.001
		NGO-managed borehole	2.17 (1.14 to 4.11)	0.02	6.93 (3.14 to 15.27)	<0.001
		Other borehole	3.77 (1.80 to 7.92)	<0.001	13.12 (5.86 to 29.37)	<0.001
Expenditure	2972	(GH₵ per day) **	0.97 (0.95 to 0.98)	<0.001	0.89 (0.87 to 0.92)	<0.001
Improved sanitation	2966	No	Reference		Reference	
		Yes	0.43 (0.31 to 0.59)	<0.001	0.28 (0.20 to 0.39)	<0.001
Soap observed	2972	No	Reference		Reference	
		Yes	0.56 (0.42 to 0.76)	<0.001	0.44 (0.33 to 0.58)	<0.001
Water obtained from source or vessel	2853	From source	Reference		Reference	
		From covered vessel	3.40 (2.48 to 4.67)	<0.001	5.62 (3.99 to 7.91)	<0.001
		From uncovered vessel	6.11 (2.56 to 14.60)	<0.001	24.29 (10.05 to 58.71)	<0.001
Urban	2972	No	Reference		Reference	
		Yes	0.43 (0.32 to 0.58)	<0.001	0.19 (0.14 to 0.26)	<0.001
Water storage container	2960	Plastic bucket or container	Reference		Reference	
		Pot or earthenware vessel	3.57 (2.23 to 5.72)	<0.001	4.35 (2.69 to 7.02)	<0.001
		Metal container	1.56 (0.81 to 2.99)	0.18	2.94 (1.60 to 5.38)	0.001
		Other (including sachets)	0.36 (0.25 to 0.53)	<0.001	0.04 (0.02 to 0.09)	<0.001
Distance between latrine and water source	2972	Within 30 m	Reference		Reference	
		More than 30 m	0.82 (0.48 to 1.39)	0.45	0.87 (0.52 to 1.45)	0.60
		Not ground water	0.20 (0.12 to 0.32)	<0.001	0.08 (0.05 to 0.12)	<0.001

* RRR = relative risk ratio. ** RRRs for expenditure relate to a GH₵1 increase in expenditure.

**Table 3:** Relative risk ratios for medium (1 to 72 cfu/100 mL) and high (>72 cfu/100 mL) contamination of point-of-consumption drinking water with *E. coli* derived from multivariable multinomial regression analysis.

Covariate	Category	Medium Contamination (1 to 72 CFU)		High Contamination (73+ CFU)	
		RRR * (95% CI)	*p*-Value	RRR * (95% CI)	*p*-Value
Constant		2.79 (1.36 to 5.74)	0.005	1.14 (0.45 to 2.94)	0.78
Water source	Piped to premises	Reference		Reference	
	Standpipe, tanker or neighbours tap	2.37 (1.17 to 4.83)	0.02	8.37 (3.44 to 20.34)	<0.001
	Protected well	0.96 (0.28 to 3.33)	0.95	9.32 (2.07 to 41.91)	0.004
	Unprotected well or spring	2.13 (0.66 to 6.90)	0.21	10.27 (2.81 to 37.46)	<0.001
	Rainwater	2.67 (0.16 to 44.15)	0.49	15.73 (0.90 to 276.53)	0.06
	Surface water	2.17 (0.73 to 6.45)	0.16	42.01 (12.73 to 138.63)	<0.001
	Packaged water	0.18 (0.09 to 0.37)	<0.001	0.04 (0.01 to 0.15)	<0.001
	Community-managed borehole	2.02 (0.97 to 4.21)	0.06	6.36 (2.49 to 16.26)	<0.001
	NGO-managed borehole	2.85 (1.01 to 8.03)	0.05	6.86 (1.78 to 26.52)	0.005
	Other borehole	1.77 (0.40 to 7.90)	0.46	8.68 (1.76 to 42.82)	0.008
Expenditure	(GH₵ per day) **	0.98 (0.95 to 1.00)	0.08	0.98 (0.94 to 1.01)	0.22
Water source * expenditure interaction	Piped to premises	Reference		Reference	
	Standpipe, tanker or neighbours tap	0.98 (0.95 to 1.01)	0.24	0.97 (0.92 to 1.02)	0.20
	Protected well	1.05 (0.94 to 1.17)	0.41	0.96 (0.84 to 1.01)	0.61
	Unprotected well or spring	1.03 (0.91 to 1.18)	0.60	1.01 (0.89 to 1.15)	0.87
	Rainwater	1.04 (0.78 to 1.40)	0.78	0.92 (0.67 to 1.25)	0.59
	Surface water	0.97 (0.89 to 1.06)	0.52	0.89 (0.82 to 0.97)	0.009
	Packaged water	1.02 (1.00 to 1.05)	0.10	1.01 (0.93 to 1.10)	0.85
	Community-managed borehole	1.01 (0.98 to 1.04)	0.43	1.00 (0.94 to 1.06)	0.97
	NGO-managed borehole	0.91 (0.82 to 1.01)	0.08	0.93 (0.82 to 1.06)	0.29
	Other borehole	1.08 (0.90 to 1.30)	0.41	1.02 (0.86 to 1.23)	0.79
Improved sanitation	No	Reference		Reference	
	Yes	0.82 (0.58 to 1.15)	0.26	0.90 (0.62 to 1.30)	0.56
Water obtained from source or vessel	From source	Reference		Reference	
	From covered vessel	0.86 (0.59 to 1.23)	0.40	0.60 (0.39 to 0.93)	0.02
	From uncovered vessel	1.86 (0.76 to 4.56)	0.17	2.82 (1.14 to 6.96)	0.03

* RRR = relative risk ratio. *N* = 2822. ** RRRs for expenditure relate to a GH₵1 increase in expenditure.

**Table 4:** Multivariable logistic regression results for detectable *E. coli* in 100 mL samples of sachet water (samples obtained directly from the source; *n* = 132).

Variable	Category	Odds Ratio (95% CI)	*p*-Value
Constant		0.12 (0.02 to 0.76)	0.03
Urban	No	Reference	
	Yes	2.26 (0.48 to 10.74)	0.30
Region	Greater Accra	Reference	
	Central	2.55 (0.58 to 11.28)	0.22
	Other	0.49 (0.13 to 1.83)	0.29
Expenditure		0.99 (0.94 to 1.05)	0.85

## Supplementary Materials

The following are available online at www.mdpi.com/1660-4601/13/3/303/s1, Table S1: Multivariable multinomial regression results, excluding observations with potential errors; Table S2. Descriptive statistics for *E. coli* contamination of water at the point of consumption, *n* = 2972. Table S3. Relative risk ratios for low (1 to 12 cfu/100 mL), medium (13 to 72 cfu/100 mL) and high (>72 cfu/100 mL) contamination of water at the point of consumption with *E. coli* derived from univariable multinomial regression analysis. Table S4. Relative risk ratios for low (1 to 12 cfu/100 mL), medium (13 to 72 cfu/100 mL) and high (>72 cfu/100 mL) contamination of drinking cup water with *E. coli* derived from multivariable multinomial regression analysis.
