# Supplementary material for: Effects of Sachet Water Consumption on Exposure to Microbe-Contaminated Drinking Water: Household Survey Evidence from Ghana
Source: Int J Environ Res Public Health. 2016 Mar 9;13(3):303. doi: 10.3390/ijerph13030303 (PMC4808966; doi:10.3390/ijerph13030303)
Supplement: Supplementary file 1 [file ijerph-13-00303-s001.pdf]

Supplementary Material: Effects of Sachet Water Consumption on Exposure to Microbe-Contaminated Drinking Water: Household Survey Evidence from Ghana

Jim Wright, Mawuli Dzodzomenyo, Nicola A. Wardrop, Richard Johnston, Allan Hill, Genevieve Aryeetey and Richard Adanu

Table S1. Multivariable multinomial regression results, excluding observations with potential errors.

Covariate	Category	Medium Contamination (1 to 72 CFU)		High Contamination (73+ CFU)	
		RRR * (95% CI)	*p*-value	RRR * (95% CI)	*p*-value
Constant		2.25 (1.03 to 4.90)	0.04	1.66 (0.62 to 4.46)	0.31
Water source	Piped to premises	Reference		Reference	
	Standpipe, tanker or neighbours tap	2.50 (1.18 to 5.26)	0.02	8.00 (3.22 to 19.89)	<0.001
	Protected well	1.58 (0.41 to 6.11)	0.51	12.32 (2.23 to 68.07)	0.004
	Unprotected well or spring	1.94 (0.43 to 8.81)	0.39	9.64 (1.97 to 47.20)	0.005
	Rainwater	4.30 (0.21 to 89.54)	0.35	14.08 (0.65 to 304.24)	0.09
	Surface water	2.68 (0.78 to 9.20)	0.12	42.06 (11.87 to 149.10)	<0.001
	Packaged water	0.17 (0.08 to 0.36)	<0.001	0.03 (0.008 to 0.13)	<0.001
	Community operated borehole	3.33 (1.53 to 7.25)	0.002	8.28 (3.12 to 21.99)	<0.001
	NGO operated borehole	3.74 (1.25 to 11.17)	0.02	6.94 (1.63 to 29.67)	0.009
	Other provider borehole	2.78 (0.46 to 16.77)	0.27	10.40 (1.61 to 67.16)	0.01
Expenditure (GH₵ per day)		0.98 (0.96 to 1.01)	0.15	0.97 (0.93 to 1.01)	0.18

Table S1. *Cont.*

Covariate	Category	Medium Contamination (1 to 72 CFU)		High Contamination (73+ CFU)	
		RRR * (95% CI)	*p*-value	RRR * (95% CI)	*p*-value
Water source * expenditure interaction	Piped to premises	Reference		Reference	
	Standpipe, tanker or neighbours tap	0.98 (0.94 to 1.01)	0.18	0.97 (0.93 to 1.02)	0.26
	Protected well	1.03 (0.93 to 1.15)	0.58	0.96 (0.83 to 1.10)	0.52
	Unprotected well or spring	1.06 (0.87 to 1.29)	0.59	1.05 (0.87 to 1.27)	0.62
	Rainwater	0.97 (0.71 to 1.35)	0.88	0.91 (0.65 to 1.28)	0.59
	Surface water	0.96 (0.87 to 1.05)	0.39	0.89 (0.82 to 0.97)	0.01
	Packaged water	1.02 (0.99 to 1.05)	0.13	1.01 (0.93 to 1.10)	0.78
	Community operated borehole	1.00 (0.98 to 1.03)	0.80	1.00 (0.93 to 1.07)	0.95
	NGO operated borehole	0.89 (0.80 to 0.99)	0.04	0.93 (0.81 to 1.07)	0.31
	Other provider borehole	1.07 (0.86 to 1.32)	0.56	1.02 (0.83 to 1.26)	0.85
Improved sanitation	No	Reference		Reference	
	Yes	0.69 (0.47 to 1.00)	0.05	0.73 (0.49 to 1.09)	0.12
Water obtained from source or vessel	From source	Reference		Reference	
	From covered vessel	1.01 (0.67 to 1.51)	0.97	0.58 (0.36 to 0.91)	0.02
	From uncovered vessel	1.55 (0.70 to 3.41)	0.28	1.93 (0.85 to 4.37)	0.12

* RRR = relative risk ratio. *n* = 2552.

Table S2. Descriptive statistics for *E. coli* contamination of water at the point of consumption, *n* = 2972.

Variable	Category	Contamination Level * (Rows Sum to 100%)			
		Uncontaminated	Low	Medium	High
Total		28.0%	20.4%	16.9%	34.7%
Source contamination risk factors					
Water source	Piped to premises	34.5%	36.7%	12.5%	16.4%
	Standpipe, tanker or neighbours tap	13.8%	17.8%	24.4%	44.0%
	Protected well	11.8%	16.2%	14.8%	57.2%
	Unprotected well or spring	12.2%	10.4%	26.5%	50.9%
	Rainwater	7.1%	25.0%	26.4%	41.6%
	Surface water	5.1%	3.7%	13.4%	77.8%
	Packaged water	68.8%	25.3%	3.8%	2.0%
	Community borehole	10.7%	21.6%	22.4%	45.2%
	NGO borehole	13.6%	16.7%	25.1%	44.6%
	Other borehole	7.9%	11.0%	31.6%	49.4%
	Missing	9.7%	49.0%	15.5%	25.9%
Distance between latrine and water source	Within 30m	8.6%	17.0%	21.3%	53.2%
	More than 30m	10.0%	14.2%	22.1%	53.7%
	Not ground water	42.5%	24.6%	13.0%	19.9%
Urban	No	15.5%	15.1%	20.9%	48.6%
	Yes	38.4%	24.8%	13.6%	23.3%
Recontamination risk factors					
Improved sanitation	No	13.6%	16.3%	21.3%	48.8%
	Yes	31.5%	21.4%	15.7%	31.5%
	Missing	6.3%	18.4%	54.6%	20.7%
Soap observed	No	19.3%	16.1%	22.6%	42.1%
	Yes	32.5%	22.6%	13.9%	30.9%
Water obtained from source or vessel	From source	47.1%	22.7%	10.4%	19.8%
	From covered vessel	17.4%	19.1%	22.4%	41.1%
	From uncovered vessel	6.4%	11.9%	15.7%	65.9%
	Missing	23.4%	29.0%	12.2%	35.5%

Table S2. *Cont.*

Variable	Category	Contamination Level * (Rows Sum to 100%)			
		Uncontaminated	Low	Medium	High
Total		28.0%	20.4%	16.9%	34.7%
Water storage container	Plastic bucket or container	24.7%	19.8%	17.9%	37.7%
	Pot or earthenware vessel	7.6%	17.6%	24.0%	50.7%
	Metal container	12.7%	11.5%	18.7%	57.1%
	Other (including sachets)	61.7%	27.1%	7.0%	4.2%
	Missing	59.8%	40.2%	0%	0%
Expenditure (GH₵)	Mean	13.3	10.0	7.8	6.5
	Median	9.0	7.8	5.7	4.9
	Range (min - max)	0.70–227.9	0.79–71.9	0.33–103.3	0.18–60.1

* Contamination levels: uncontaminated (0 cfu/100 mL); low contamination (1 to 12 cfu/100 mL); medium contamination (1 to 72 cfu/100 mL); high contamination (>72 cfu/100 mL).

Table S3. Relative risk ratios for low (1 to 12 cfu/100 mL), medium (13 to 72 cfu/100 mL) and high (>72 cfu/100 mL) contamination of water at the point of consumption with *E. coli* derived from univariable multinomial regression analysis.

Covariate	Category	Low Contamination (1 to 12 CFU)		Medium Contamination (13 to 72 CFU)		High Contamination (73+ CFU)	
		RRR * (95% CI)	*p*-value	RRR *(95% CI)	*p*-value	RRR *(95% CI)	*p*-value
Water source	Piped to premises	Reference		Reference		Reference	
	Standpipe, tanker or neighbours tap	1.21 (0.67 to 2.17)	0.53	4.88 (2.56 to 9.32)	<0.001	6.69 (3.57 to 12.55)	<0.001
	Protected well	1.30 (0.46 to 3.67)	0.62	3.49 (1.24 to 9.80)	0.02	10.24 (4.30 to 24.39)	<0.001
	Unprotected well or spring	0.80 0.22 to 2.93)	0.73	5.99 (1.77 to 20.24)	0.004	8.76 (2.67 to 28.76)	<0.001
	Rainwater	3.32 (0.81 to 13.66)	0.10	10.33 (2.73 to 39.05)	0.001	12.38 (3.13 to 48.94)	<0.001
	Surface water	0.68 (0.26 to 1.80)	0.44	7.31 (2.88 to 18.60)	<0.001	32.28 (13.63 to 76.48)	<0.001
	Packaged water	0.35 (0.20 to 0.59)	<0.001	0.15 (0.07 to 0.32)	<0.001	0.06 (0.03 to 0.13)	<0.001
	Community-managed borehole	1.90 (1.01 to 3.57)	0.05	5.79 (2.89 to 11.60)	<0.001	8.89 (4.68 to 16.88)	<0.001
	NGO-managed borehole	1.16 (0.55 to 2.47)	0.70	5.13 (2.42 to 10.86)	<0.001	6.93 (3.14 to 15.27)	<0.001
	Other borehole	1.31 (0.59 to 2.88)	0.50	11.03 (4.64 to 26.25)	<0.001	13.12 (5.86 to 29.37)	<0.001
Expenditure	(GH₵ per day)**	0.98 (0.97 to 0.99)	0.001	0.94 (0.92 to 0.96)	<0.001	0.89 (0.87 to 0.92)	<0.001

Table S3. *Cont.*

Covariate	Category	Low Contamination (1 to 12 CFU)		Medium Contamination (13 to 72 CFU)		High Contamination (73+ CFU)	
		RRR * (95% CI)	*p*-value	RRR *(95% CI)	*p*-value	RRR *(95% CI)	*p*-value
Improved sanitation	No	Reference		Reference		Reference	
	Yes	0.57 (0.40 to 0.81)	0.002	0.32 (0.22 to 0.46)	<0.001	0.28 (0.20 to 0.39)	<0.001
Soap observed	No	Reference		Reference		Reference	
	Yes	0.84 (0.60 to 1.17)	0.30	0.37 (0.26 to 0.52)	<0.001	0.44 (0.33 to 0.58)	<0.001
Water obtained from source or vessel	From source	Reference		Reference		Reference	
	From covered vessel	2.28 (1.60 to 3.25)	<0.001	5.86(3.80 to 9.02)	<0.001	5.62 (3.99 to 7.91)	<0.001
	From uncovered vessel	3.83 (1.48 to 9.90)	0.006	11.10 (4.39 to 28.07)	<0.001	24.29 (10.05 to 58.71)	<0.001
Urban	No	Reference		Reference		Reference	
	Yes	0.66 (0.47 to 0.93)	0.02	0.26 (0.18 to 0.38)	<0.001	0.19(0.14 to 0.26)	<0.001
Water storage container	Plastic bucket or container	Reference		Reference		Reference	
	Pot or earthenware vessel	2.88 (1.65 to 5.02)	<0.001	4.34(2.62 to 7.18)	<0.001	4.35 (2.69 to 7.02)	<0.001
	Metal container	1.13 (0.53 to 2.42)	0.76	2.04(1.00 to 4.15)	0.05	2.94 (1.60 to 5.38)	0.001
	Other (including sachets)	0.55(0.36 to 0.82)	0.004	0.16 (0.09 to 0.29)	<0.001	0.04 (0.02 to 0.09)	<0.001
Distance between latrine and water source	Within 30m	Reference		Reference		Reference	
	More than 30m	0.72 (0.39 to 1.32)	0.28	0.90 (0.52 to 1.55)	0.70	0.87 (0.52 to 1.45)	0.60
	Not ground water	0.29 (0.18 to 0.49)	<0.001	0.12 (0.07 to 0.31)	<0.001	0.08 (0.05 to 0.12)	<0.001

* RRR = relative risk ratio. ** RRRs for expenditure relate to a GH₵1 increase in expenditure.

Table S4. Relative risk ratios for low (1 to 12 cfu/100 mL), medium (13 to 72 cfu/100 mL) and high (>72 cfu/100 mL) contamination of drinking cup water with *E. coli* derived from multivariable multinomial regression analysis.

Covariate	Category	Low Contamination (1 to 12 CFU)		Medium Contamination (13 to 72 CFU)		High Contamination (73+ CFU)	
		RRR * (95% CI)	*p*-Value	RRR * (95% CI)	*p*-Value	RRR * (95% CI)	*p*-Value
Constant		2.10 (0.99 to 4.43)	0.05	0.69 (0.27 to 1.77)	0.44	1.17 (0.45 to 3.04)	0.74
Water source	Piped to premises	Reference		Reference		Reference	
	Standpipe, tanker or neighbours tap	1.22 (0.56 to 2.63)	0.61	6.26 (2.49 to 15.73)	<0.001	8.40 (3.44 to 20.52)	<0.001
	Protected well	0.36 (0.10 to 1.30)	0.12	5.52 (0.88 to 34.54)	0.07	9.91 (1.93 to 51.02)	0.006
	Unprotected well or spring	0.94 (0.22 to 3.97)	0.93	6.19 (1.65 to 23.25)	0.007	10.28 (2.81 to 37.66)	<0.001
	Rainwater	0.85 (0.04 to 16.12)	0.91	10.73 (0.53 to 219.40)	0.12	16.17 (0.87 to 299.05)	0.06
	Surface water	0.91 (0.24 to 3.38)	0.88	6.94 (2.00 to 24.04)	0.002	42.52 (12.78 to 141.43)	<0.001
	Packaged water	0.20 (0.10 to 0.41)	<0.001	0.12 (0.04 to 0.38)	<0.001	0.04 (0.009 to 0.15)	<0.001
	Community-managed borehole	1.25 (0.58 to 2.72)	0.57	4.54 (1.75 to 11.82)	0.002	6.36 (2.48 to 16.31)	<0.001
	NGO-managed borehole	1.07 (0.30 to 3.86)	0.91	10.13 (3.25 to 31.54)	<0.001	6.95 (1.78 to 27.14)	0.005
	Other borehole	0.79 (0.16 to 3.97)	0.78	5.25 (1.04 to 26.46)	0.05	8.68 (1.74 to 43.27)	0.008
Expenditure	(GH₵ per day) **	0.97 (0.94 to 1.00)	0.03	0.99 (0.96 to 1.02)	0.54	0.98 (0.94 to 1.02)	0.22
Water source * expenditure interaction	Piped to premises	Reference		Reference		Reference	
	Standpipe, tanker or neighbours tap	0.98 (0.94 to 1.02)	0.40	0.97 (0.93 to 1.01)	0.15	0.97 (0.92 to 1.02)	0.21
	Protected well	1.10 (0.98 to 1.24)	0.10	0.90 (0.75 to 1.08)	0.27	0.96 (0.82 to 1.12)	0.59
	Unprotected well or spring	1.02 (0.89 to 1.17)	0.74	1.03 (0.90 to 1.18)	0.68	1.01 (0.89 to 1.15)	0.86
	Rainwater	1.12 (0.83 to 1.53)	0.46	0.96 (0.70 to 1.32)	0.81	0.92 (0.67 to 1.26)	0.59
	Surface water	0.91 (0.80 to 1.04)	0.17	0.97 (0.88 to 1.06)	0.51	0.89 (0.82 to 0.97)	0.009
	Packaged water	1.03 (1.00 to 1.06)	0.04	1.00 (0.95 to 1.05)	0.89	1.01 (0.93 to 1.10)	0.85
	Community-managed borehole	1.02 (0.99 to 1.05)	0.20	1.00(0.96 to 1.04)	0.87	1.00 (0.94 to 1.06)	0.98
	NGO-managed borehole	0.97 (0.85 to 1.09)	0.58	0.84 (0.75 to 0.95)	0.004	0.93 (0.82 to 1.06)	0.29
	Other borehole	1.04 (0.87 to 1.25)	0.65	1.08 (0.89 to 1.31)	0.44	1.03 (0.85 to 1.23)	0.79

Table S4. *Cont.*

Covariate	Category	Low Contamination (1 to 12 CFU)		Medium Contamination (13 to 72 CFU)		High Contamination (73+ CFU)	
Improved sanitation	No	Reference		Reference		Reference	
	Yes	0.85 (0.58 to 1.23)	0.38	0.80 (0.53 to 1.21)	0.29	0.89 (0.61 to 1.30)	0.54
Water obtained from source or vessel	From source	Reference		Reference		Reference	
	From covered vessel	0.90 (0.61 to 1.33)	0.60	0.78 (0.46 to 1.33)	0.37	0.59 (0.38 to 0.92)	0.02
	From uncovered vessel	1.95 (0.73 to 5.19)	0.18	1.69 (0.67 to 4.28)	0.27	2.73 (1.13 to 6.23)	0.03

* RRR = relative risk ratio. N = 2822. ** RRRs for expenditure relate to a GH₵1 increase in expenditure.

© 2016 by the authors; licensee MDPI, Basel, Switzerland. This article is an open access article distributed under the terms and conditions of the Creative Commons by Attribution (CC-BY) license (http://creativecommons.org/licenses/by/4.0/).
